# A novel approach for nitrogen diagnosis of wheat canopies digital images by mobile phones based on histogram

**DOI:** 10.1038/s41598-021-92431-5

**Published:** 2021-06-21

**Authors:** Xin Qi, Yanan Zhao, Yufang Huang, Yang Wang, Wei Qin, Wen Fu, Yulong Guo, Youliang Ye

**Affiliations:** 1 College of Resources and Environment, Henan Agricultural University, Zhengzhou, 450002 China; 2 Agriculture Green Development Engineering & Technology Center, Zhengzhou, 450002 China; 3 College of Resources and Environment, National Academy of Agricultural Green Development, China Agricultural University, Beijing, 100193 China

**Keywords:** Plant sciences, Environmental sciences

## Abstract

The accurate and nondestructive assessment of leaf nitrogen (N) is very important for N management in winter wheat fields. Mobile phones are now being used as an additional N diagnostic tool. To overcome the drawbacks of traditional digital camera diagnostic methods, a histogram-based method was proposed and compared with the traditional methods. Here, the field N level of six different wheat cultivars was assessed to obtain canopy images, leaf N content, and yield. The stability and accuracy of the index histogram and index mean value of the canopy images in different wheat cultivars were compared based on their correlation with leaf N and yield, following which the best diagnosis and prediction model was selected using the neural network model. The results showed that N application significantly affected the leaf N content and yield of wheat, as well as the hue of the canopy images and plant coverage. Compared with the mean value of the canopy image color parameters, the histogram could reflect both the crop coverage and the overall color information. The histogram thus had a high linear correlation with leaf N content and yield and a relatively stable correlation across different growth stages. Peak b of the histogram changed with the increase in leaf N content during the reviving stage of wheat. The histogram of the canopy image color parameters had a good correlation with leaf N content and yield. Through the neural network training and estimation model, the root mean square error (RMSE) and the mean absolute percentage error (MAPE) of the estimated and measured values of leaf N content and yield were smaller for the index histogram (0.465, 9.65%, and 465.12, 5.5% respectively) than the index mean value of the canopy images (0.526, 12.53% and 593.52, 7.83% respectively), suggesting a good fit for the index histogram image color and robustness in estimating N content and yield. Hence, the use of the histogram model with a smartphone has great potential application in N diagnosis and prediction for wheat and other cereal crops.

## Introduction

Optimal nitrogen (N) fertilization is important for crop growth and yield. While N deficiency may result in small leaves with a low chlorophyll content and reduced biomass, and thus yield^1^, excessive N application may lead to a low N use efficiency (NUE) as well as ecological and environmental problems^2^.

The convenient and accurate diagnosis of crop N nutrition can improve NUE through real-time N management. At present, there are many methods for N diagnosis, such as soil inorganic N, plant N concentration, and plant nitrate. Instruments for N diagnosis include a chlorophyll meter, spectrometer, unmanned aerial vehicle, and digital camera^3–8^. However, the above methods and instruments have some limitations in N diagnosis, as they are time-consuming, destructive, and expensive^9,10^. Barbedo found that close-range images can be used to detect visual changes in plant color and morphology and machine learning techniques become an effective solution^11^. Diagnosing crops without causing damage is important for optimizing N management.

N nutrition diagnosis using a mobile phone camera is essentially the application of digital image technology. Analyses of RGB images of digital cameras can help to evaluate the chlorophyll content of leaves as well as the growth of crops, these two parameters are closely related to N content and crop yield, respectively. In recent years, many studies have focused on the application of digital images in the diagnosis of crop N nutrition, for example, in maize, wheat, rice, and other crops^12–23^. Recently, Yuttana et al.^24^ showed that mobile phone cameras could be used as analyzers to detect the color of rice leaves. Zhang et al.^12^ found that when using digital cameras to diagnose N nutrition in summer maize, the 6-leaf stage was considered as a key period. Xiao et al.^25^ found that the R/(R + G + B) of digital images of wheat could be highly correlated with conventional nutritional diagnostic indicators, such as chlorophyll, nitrate concentration at the base of the stem, and total N content of the plant at the jointing stage. Moreover, the normalized redness intensity (NRI) and CMI (color mix index, a*R + b*G + C*b) were also suitable as characteristic parameters of wheat N nutrition diagnosis^26^.

At present, many technical problems still need to be resolved for improved N nutrition diagnosis using mobile cameras. Generally, the mean value of various image color indexes is calculated and combined with traditional diagnostic parameters in regression models; this mean value, however, only reflects the overall color status of the entire study area. The better the crop grows, the greater the proportion in the image. More detailed information that can reflect the nutrition level is missing, such as the leaf growth and the color difference. Different leaf types can affect the size of the leaf area. Moreover, differences in leaf color and leaf type among different wheat cultivars may affect the color parameters of the crop canopy image, thus affecting the N nutrition diagnosis results of the digital images and the application of diagnosis technology. It is important for model stability to study the differences in individual nutritional diagnoses among different wheat cultivars. However, there is a lack of comparative studies among different wheat cultivars. In the present study, RGB images of the canopies of six wheat cultivars (‘Huayu 198’, ‘Yumai 49-198’, ‘Zhongmai 1’, ‘Xinong 979’, ‘Pingan 8’, and ‘Taixue 12’) were taken during the reviving and jointing stages using a standard mobile phone camera.

The image histogram, a classic image statistical feature, is widely used in digital image analysis^27–29^. It uses a curve in the pixel value-pixel number (or percentage) space to describe the color distribution of the current image. Due to the number of pixels at each data range, a histogram can offer much more information than the mean value of the image. Thus, a histogram offers a novel way to connect winter wheat N nutrition and its canopy digital image. The extraction and effective use of the information extracted from the canopy digital images requires comprehensive discussion.

Therefore, the objectives of the study were (i) to evaluate the cultivar sensitivity of the index image mean value (IIMV) and index image histogram (IIH) to leaf N content and yield using six wheat cultivars; and (ii) to build the N nutrition and yield diagnostic models based on the neural network algorithm. Then, the performance of the IIMV models and IIH models was compared to select the best diagnostic model, providing theoretical support for the precision fertilization of wheat.

## Materials and methods

### Ethics statement

All samples come from field trials conducted by the research group, and the sampling and operation of the trials comply with legal and ethical requirements.

### Experimental design

The study was conducted in Kangcheng, Shundian (3427ʹ N, 11,336ʹ E), Yuzhou, Province Henan, in East-central China from October 2017 to June 2018. The soil type was aquic brown soil, the soil texture was clay soil, and the previous crop was maize. The soil pH (0–20 cm) before sowing was 8.2, the organic matter was 20.5 g·kg^−1^, the total N was 0.92 g·kg^−1^, the nitrate N was 29.48 mg·kg^−1^, the available phosphorus was 22.0 mg·kg^−1^, and the available potassium was 135.7 mg·kg^−1^. The wheat cultivars included Huayu 198 (HY198), Yumai 49-198 (YM49-198), Zhongmai 1 (ZM1), Xinong 979 (XN979), Ping'an 8 (PA8), and Taixue 12 (TX12). Five N treatments were used, including 0, 120, 180, 240, and 360 kg N ha^−1^ (N0, N120, N180, N240, and N360, respectively), and were provided as urea and applied twice, with 50% as the base application and 50% topdressing during the jointing stage. Phosphorus and potassium (kg ha^−1^) were applied once as calcium superphosphate and potassium chloride on plots of 40 m^2^, which were arranged in random blocks, with three repetitions. The same amount of wheat (180 kg ha^−1^) was sown on each plot on October 13, 2017, and was harvested on June 5, 2018.

### Measurement items and methods

Determination of the N concentration of the plants and leaves: five plants from each plot were sampled and then separated into leaves and stems. The samples were pre-dried at 105 °C for 30 min and then dried to a constant mass at 70 °C in an oven to determine the dry mass (DM). The plant materials were ground to pass through a 2-mm mesh screen, and aliquots were ground for further analyses. The samples were digested with H_2_SO_4_ and H_2_O_2_, and the total N concentration of the digested samples was determined using an automated continuous flow analyzer (Seal, Norderstedt, Germany). In the wheat harvesting period, 5 m^2^ wheat was artificially harvested in the production area of each plot, and the yield was counted after drying.

### Crop canopy image acquisition method

The shooting stage includes the reviving stage and jointing stage of winter wheat. Photographs were taken between 12:00 and 14:00 on cloudless days. Smartphones were used to obtain the wheat canopy image. The shooting height was 1.2 m above the ground.

### Crop canopy image processing and color parameter design

Mobile digital images have three color channels, including R, G, and B. This can capture the vegetation canopy’s reflected light characteristics of the three brands (red, green, and blue), which are directly related to the vegetation absorption characteristics. Chlorophyll is the most important pigment in wheat and has strong absorption of blue and red light, but less absorption of green light^30^. Based on this basic characteristic and the crop canopy image, ENVI (ENVI 5.0, ESIR, USA) was used to calculate six color indices, namely G/R, G/B, R/(R + G + B), G/(R + G + B), B/(R + G + B), and (G-R)/(R + G + B).

Among them, G/R is the ratio of the green light channel image to the red channel. The higher the green channel, the lusher the vegetation, and the smaller the red channel, the more the chlorophyll. G/R can highlight vegetation information^31^. The G/B ratio is similar to G/R, in that the higher the ratio, the more chlorophyll the vegetation has^32^. In R/(R + G + B), the greater the proportion of red light in the entire image, the less the chlorophyll content of the vegetation^32^, while in G/(R + G + B), the higher the green channel, the lusher the vegetation^33^. In B/(R + G + B), the larger the red light channel, the less the vegetation^34^, and (G-R)/(R + G + B) is the subtraction between the green and red channels and thus highlights the vegetation information and reflects the plant status.

**Figure 1:**
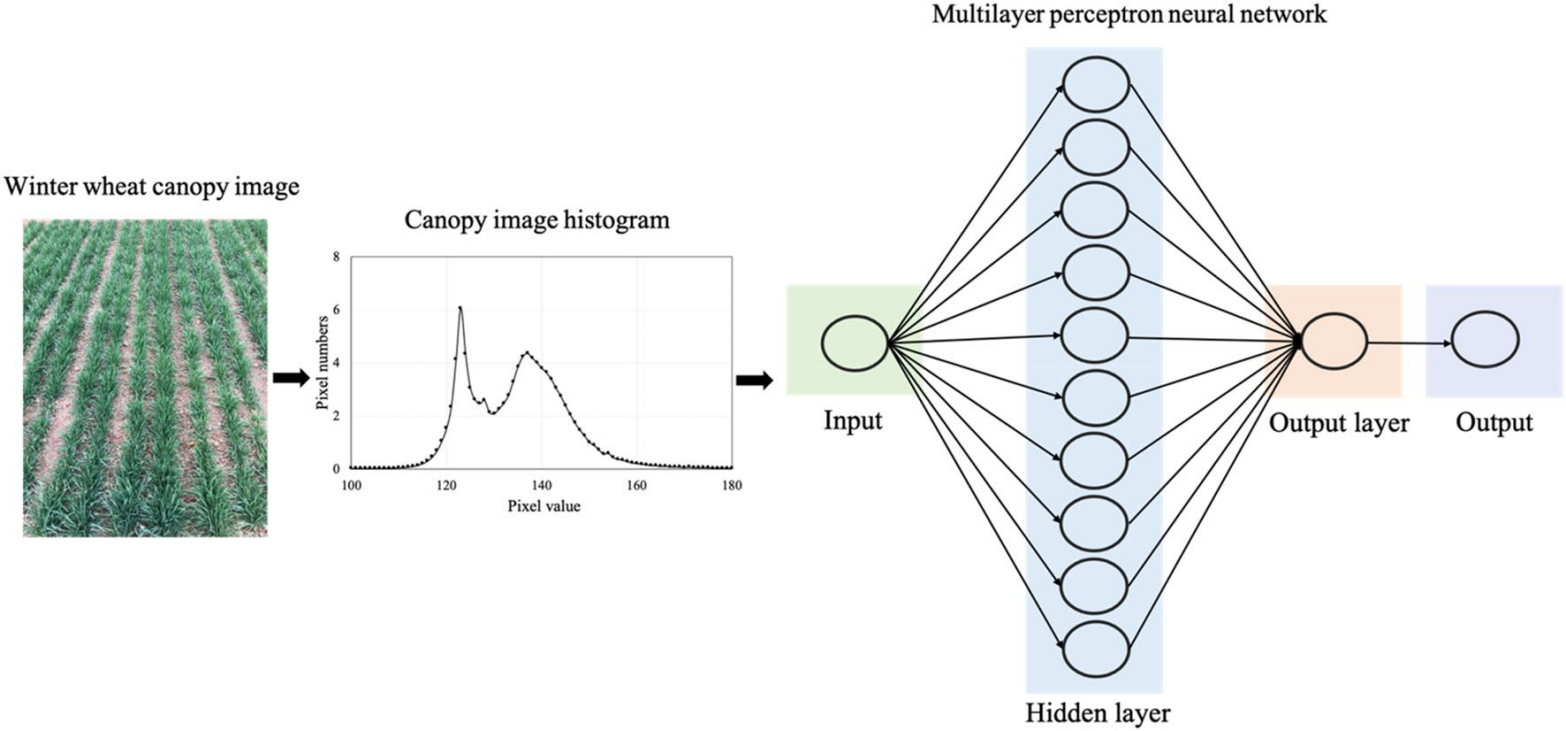
Schematic diagram of winter wheat canopy image processing and analysis methods.

The color index image is converted into an independent variable in two ways. The first method takes the mean value of the vegetation component in the image as the model independent variable to construct a single-variable diagnostic approach; the second method calculates the histogram of the color index images. Correlation analysis between histogram curves and leaf N concentration and yield was used to filter highly interrelated curve regions. The selected histogram region was set as independent variables, and a multi-variable diagnostic approach was constructed. The color index, the mean value of the index image, and the histogram of the index image were calculated by ENVI software. The specific histogram-based method used in this study is shown in Fig. 1.

### Neural network diagnostic model design

**Figure 2:**
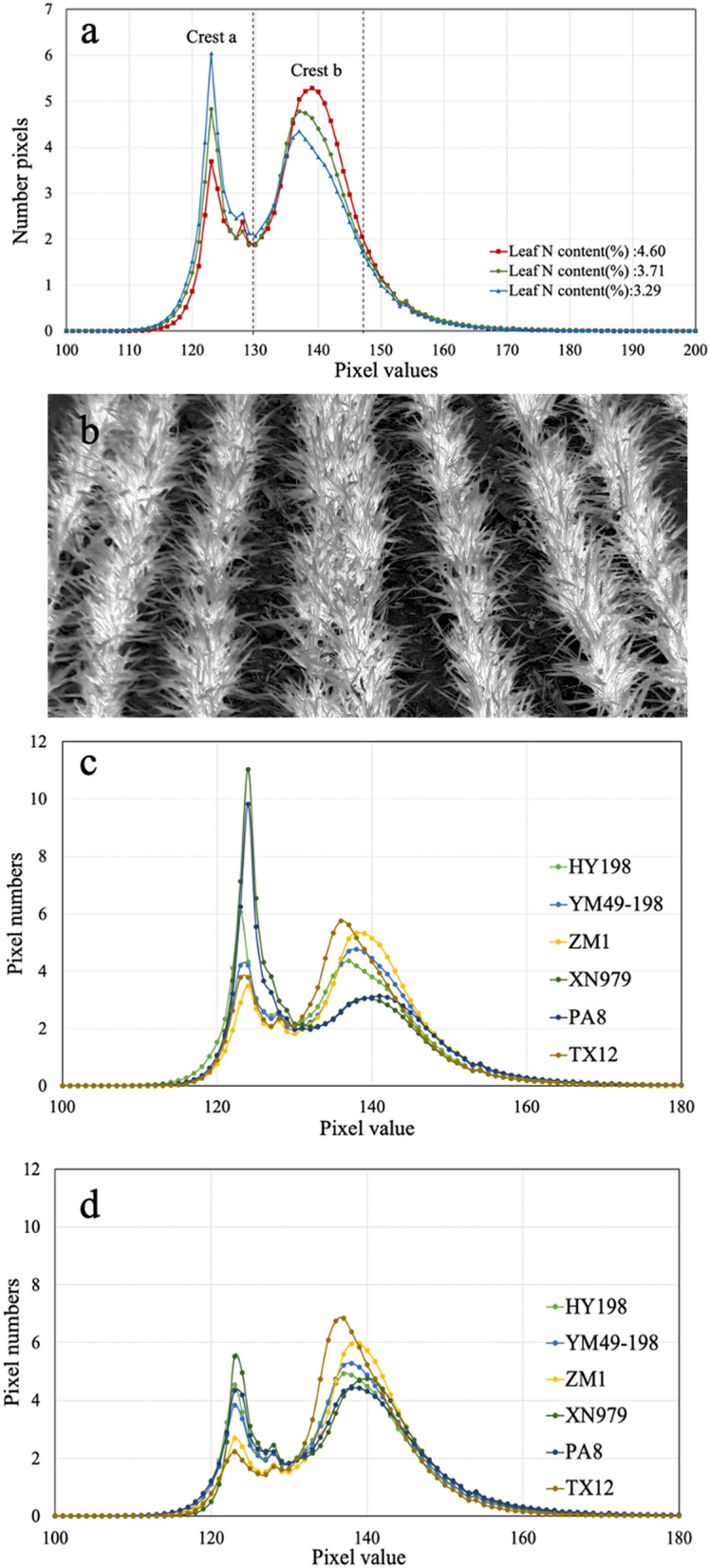
Histogram of wheat at the reviving stage (the histogram uses [(G-R)/(R + G + B)] as an example). (**a**) At different leaf N contents (HY198 as an example), the areas within the two dashed lines were used as the data for the correlation analysis of the histogram and the leaf N content and yield. (**c**) Different wheat cultivars at minimum leaf N content (minimum leaf N content of different cultivars). (**d**) Different wheat cultivars at maximum leaf N content (maximum leaf N content of different cultivars).

In order to compare the adaptability of IIMV and IIH to different wheat cultivars, a neural network was used to combine the data of the six cultivars. The relationship between the filtered IIH segment and its dependent variable, i.e., the diagnostic factor, cannot be easily explained by a simple linear relationship. Therefore, an artificial neural network was used for fitting. A neural network model is a highly non-linear model that performs well in complex nonlinear problems^35^. The neural network model (multilayer perceptron neural networks) of MATLAB (R2015b, MathWorks, USA) was used to create and train a network by using a neural network fitting tool. There were 1 hidden layer with 10 neurons and 1 output layer with 1 neuron in our network. The transfer functions of the 2 layers were sigmoid and linear, respectively. The Levenberg–Marquardt backpropagation algorithm was used in weight optimizing. The independent variables near the peak value (Fig. 2a) were selected from the histogram of the color parameters of the canopy image for neural network training. For comparative analysis, the neural network model was used to train and fit the mean value of the image color parameters with the independent variables, and then the optimal result was selected.

In artificial neural network simulation, through repeated comparisons and trials, LM (Levenberg–Marquardt) neural net fitting in MATLAB is often used as a training method. Canopy images of winter wheat microscopical performance in the field were collected and used in conjunction with the optimal histogram section information corresponding to the optimal color index as the input variable of the neural network. The leaf N content and yield corresponding to the image were used as the output variables. The training results were obtained by fitting the data of the reviving stage and jointing stage with the leaf N content and yield. There were 90 samples in each stage and 15 samples per cultivar per stage, and three images were used for each N-fertilizer level. Sixty samples were used for training, and the other 30 samples were used to test the training effect to ensure the stability and accuracy of the training and then obtain the network. The results produced by neural network learning were used as part of the system knowledge to achieve a non-destructive N nutrition diagnosis of wheat.

## Results

### Effects of N fertilizer application on the N nutrition and yield of different wheat cultivars

**Figure 3:**
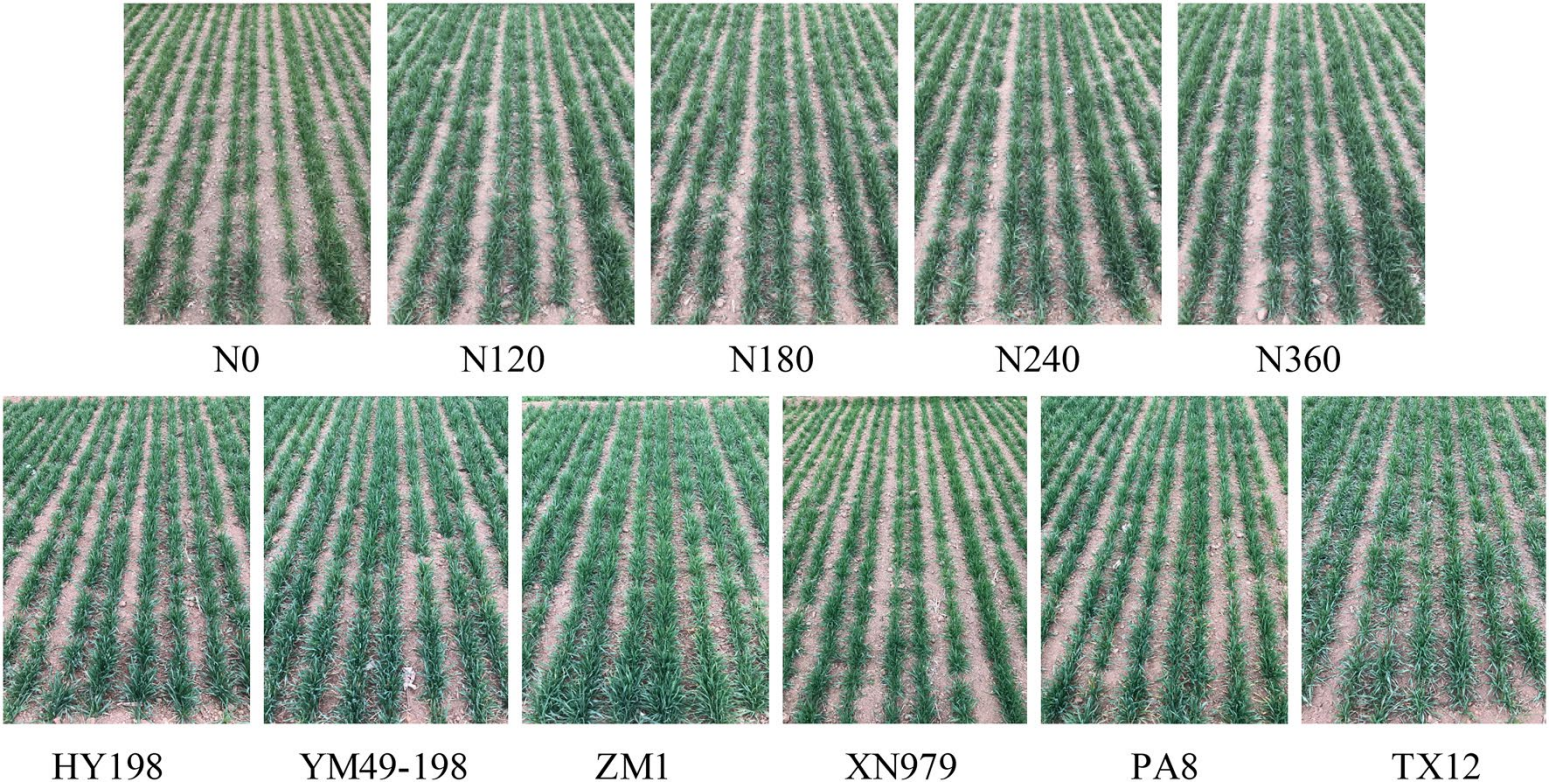
Canopy images of wheat with five N-fertilizer application levels (XN979 as an example); Canopy image of different cultivars under same N-fertilizer application levels (N0 as an example).

From the RGB images of the canopies (Fig. 3), it is obvious that wheat plants of the same cultivar, but grown without N application, were yellowish-green and grew less vigorously, while the plants grown at the various N concentrations did not differ visually in these parameters.

At the reviving stage, N application significantly increased the N content of the wheat leaves (Table 1) by 16.4%, 14.5%, 11.9%, 18.7%, and 18% compared to plants not fertilized with N. At the jointing stage, the leaf N content of each cultivar increased more significantly under the different N treatments, and the average increase was 40.7%, 33.9%, 22.2%, 44.6%, 23%, and 30.1%, respectively.

**Table 1:** Leaf N content and yield of wheat in different wheat cultivars. N0, N120, N180, N240, and N360 represent N application rates of 0, 120, 180, 240, and 360 kg N ha-^−1^, respectively. CV is the coefficient of variation. Different lowercase letters indicate significant differences at the 5% probability level.

Cultivar	Nitrogen levels	Leaf N content (%)		Yield (kg ha^-1^)
		Reviving stage	Jointing stage	
Huayu 198 (HY198)	N0	3.33c	2.85c	6696b
	N120	3.51c	3.66b	7000b
	N180	3.82b	4.28a	7374a
	N240	3.86b	3.8b	6878b
	N360	4.32a	4.3a	6911b
	Mean	3.77	3.78	6972
	CV	3.07	7.09	2.73
Yumai 49–198 (YM49-198)	N0	3.19b	3.34b	6312b
	N120	3.67ab	3.9ab	6926a
	N180	3.83a	3.18b	6759a
	N240	3.97a	3.8ab	6224b
	N360	3.78ab	4.39a	5934b
	Mean	3.69	3.72	6431
	CV	7.37	8.84	3.21
Zhongmai 1 (ZM1)	N0	3.19b	2.78a	6351c
	N120	3.66ab	3.7a	6555bc
	N180	3.83ab	3.67a	6878ab
	N240	3.42ab	3.04a	7054a
	N360	4.00a	3.52a	6827ab
	Mean	3.60	3.34	6733
	CV	11.80	8.78	3.73
Xinong 979 (XN979)	N0	2.34b	2.89b	3549d
	N120	3.97a	3.85a	5657c
	N180	3.63ab	4.25a	6862a
	N240	3.76ab	4.11a	6167b
	N360	4.45a	4.28a	5918bc
	Mean	3.63	3.88	5631
	CV	17.34	8.65	4.32
Pingan 8 (PA8)	N0	3.2bc	2.67b	6474b
	N120	2.84 cd	3.11b	6474b
	N180	2.77d	3.00b	6934a
	N240	3.69a	3.95a	7004a
	N360	3.54ab	3.96a	7013a
	Mean	3.21	3.34	6763
	CV	7.00	7.55	2.96
Taixue 12 (TX12)	N0	3.36a	3.56abc	5973ab
	N120	3.98a	3.65abc	5890b
	N180	3.96a	3.83a	6122ab
	N240	3.76a	3.83ab	6291a
	N360	4.02a	3.52abc	5976ab
	Mean	3.81	3.68	6050
	CV	11.84	2.76	3.23

Different lowercase letters indicate statistically significant differences between different treatments at P < 0.05 level.

Irrespective of the cultivar, grain yield was maximum for plants that were N-fertilized at 120 and 180 kg ha^−1^ (Table 1). However, there were some minor differences between the yield responses of the plants of some cultivars, i.e., the plants of ‘HY198’ performed well even without additional N, while those of Zhongmai 1 (ZM1) and Ping'an 8 (PA8) had maximal yields at N240 and N360, respectively. The results suggest that appropriate N application can increase wheat yield, but that too much N application may decrease yield.

### Mean value of the canopy image color parameters and its relationship with leaf N content and yield

Tables 2 and 3 show the mean value of the different color parameters under different N fertilizer treatments during the reviving stage and jointing stage, respectively. The R, G, and B light values were in the order of G > B > R during the reviving stage and G > R > B during the jointing period. The mean values of the color parameters G/R and [(G-R)/(R + G + B)] were relatively stable in the different wheat growth stages. In the different cultivars, the mean value of the non-N-fertilized color parameters was significantly different from that of the other N-fertilized treatments.

**Table 2:** Mean values of the color parameters of canopy images of wheat at the reviving stage.

Cultivar	Nitrogen levels	Color parameter					
		G/R	G/B	NRI	NGI	NBI	G-R/R + G + B
Huayu 198 (HY198)	N0	1.206b	1.128b	0.313a	0.364b	0.324b	0.051b
	N120	1.258a	1.132ab	0.304b	0.368a	0.327ab	0.064a
	N180	1.267a	1.138ab	0.303b	0.370a	0.327ab	0.067a
	N240	1.251a	1.151a	0.309b	0.369a	0.325ab	0.064a
	N360	1.265a	1.134ab	0.303b	0.369a	0.328a	0.066a
	Mean	1.249	1.136	0.306	0.368	0.326	0.062
	CV	1.264	0.914	0.893	0.522	0.608	7.201
Yumai 49-198 (YM49-198)	N0	1.262a	1.134a	0.304a	0.353a	0.327b	0.065b
	N120	1.256a	1.125ab	0.304a	0.368a	0.328ab	0.064b
	N180	1.261a	1.117b	0.302ab	0.368a	0.330a	0.065ab
	N240	1.275a	1.126ab	0.300b	0.370a	0.330a	0.070ab
	N360	1.283a	1.130ab	0.301b	0.371a	0.329a	0.071a
	Mean	1.267	1.126	0.302	0.366	0.329	0.067
	CV	1.215	0.711	0.554	0.315	2.048	4.544
Zhongmai 1 (ZM1)	N0	1.309b	1.162a	0.298a	0.376a	0.326a	0.078b
	N120	1.311b	1.153a	0.297a	0.375a	0.327a	0.078b
	N180	1.355a	1.168a	0.292b	0.368a	0.328a	0.089a
	N240	1.334ab	1.163a	0.294ab	0.378a	0.327a	0.084ab
	N360	1.333ab	1.175a	0.300a	0.380a	0.325a	0.084ab
	Mean	1.329	1.164	0.296	0.376	0.326	0.083
	CV	1.574	1.102	0.871	0.622	1.626	5.030
Xinong 979 (XN979)	N0	1.180b	1.206a	0.323a	0.368b	0.309b	0.045b
	N120	1.305a	1.167b	0.301b	0.375a	0.324a	0.074a
	N180	1.291a	1.168b	0.303b	0.375a	0.323a	0.072a
	N240	1.282a	1.164b	0.303b	0.374a	0.323a	0.070a
	N360	1.282a	1.157b	0.304b	0.372a	0.324a	0.069a
	Mean	1.268	1.173	0.307	0.373	0.320	0.066
	CV	1.354	0.796	0.783	0.394	0.615	6.942
Pingan 8 (PA8)	N0	1.245b	1.177ab	0.311a	0.371ab	0.318bc	0.060c
	N120	1.247b	1.205a	0.312a	0.375a	0.314c	0.063bc
	N180	1.249b	1.205a	0.312a	0.374ab	0.314c	0.062bc
	N240	1.272a	1.137b	0.303b	0.370b	0.327a	0.067ab
	N360	1.293a	1.147b	0.302b	0.372ab	0.326ab	0.070a
	Mean	1.261	1.174	0.308	0.372	0.320	0.064
	CV	0.930	2.121	0.921	1.128	0.634	4.591
Taixue 12 (TX12)	N0	1.235c	1.154a	0.315a	0.369b	0.322a	0.060b
	N120	1.290a	1.172a	0.302b	0.375a	0.323a	0.074a
	N180	1.276ab	1.170a	0.303b	0.374a	0.323a	0.071a
	N240	1.265abc	1.170a	0.305b	0.374a	0.322a	0.070a
	N360	1.245bc	1.158a	0.304ab	0.372ab	0.323a	0.068ab
	Mean	1.262	1.165	0.306	0.373	0.323	0.068
	CV	1.549	0.850	1.540	0.416	0.646	6.513

Note: Different lowercase letters indicate statistically significant differences between different treatments at P < 0.05 level.

**Table 3:** Mean values of the color parameters of canopy images of wheat at the jointing stage.

Cultivar	Nitrogen levels	Color parameter					
		G/R	G/B	NRI	NGI	NBI	G-R/R + G + B
Huayu 198 (HY198)	N0	1.574a	4.579c	2.177a	3.114a	1.181a	0.174a
	N120	1.581a	4.855b	2.157ab	3.116a	1.142ab	0.177a
	N180	1.578a	4.748bc	2.163a	3.107a	1.159a	0.176a
	N240	1.559a	5.171a	2.115b	3.030b	1.068c	0.173a
	N360	1.564a	4.953ab	2.159ab	3.096a	1.109bc	0.174a
	Mean	1.571	4.861	2.154	3.093	1.132	0.175
	CV	0.867	2.625	1.035	1.092	2.066	1.772
Yumai 49–198 (YM49-198)	N0	1.531b	4.565b	2.240a	3.153a	1.143a	0.166ab
	N120	1.541b	4.968a	2.237a	3.151a	1.075b	0.169ab
	N180	1.550ab	4.648b	2.211a	3.127a	1.116ab	0.170a
	N240	1.532b	4.489b	2.218a	3.110a	1.135a	0.165b
	N360	1.565a	4.546b	2.205a	3.128a	1.133a	0.169ab
	Mean	1.544	4.643	2.222	3.134	1.120	0.168
	CV	0.631	2.643	1.099	1.071	2.351	1.247
Zhongmai 1 (ZM1)	N0	1.525a	3.915ab	2.261a	3.158ab	1.245a	0.163a
	N120	1.500b	3.958ab	2.253a	3.121ab	1.230a	0.157b
	N180	1.511ab	3.728bc	2.216a	3.081b	1.254a	0.158b
	N240	1.499b	3.626c	2.291a	3.164ab	1.303a	0.155b
	N360	1.523a	4.061a	2.303a	3.214a	1.261a	0.163a
	Mean	1.512	3.857	2.265	3.148	1.259	0.159
	CV	0.591	3.728	2.344	1.931	3.160	1.267
Xinong 979 (XN979)	N0	1.544a	4.385a	2.230b	3.109b	1.141b	0.167a
	N120	1.533a	4.589a	2.236b	3.109b	1.123b	0.166a
	N180	1.522a	4.424a	2.265b	3.145b	1.156b	0.163a
	N240	1.373b	4.984a	2.867a	3.767a	1.303ab	0.125b
	N360	1.339b	4.642a	2.911a	3.760a	1.356a	0.114b
	Mean	1.462	4.605	2.502	3.378	1.216	0.147
	CV	1.808	6.398	4.413	3.214	6.799	6.011
Pingan 8 (PA8)	N0	1.468b	5.581 a	2.364 a	3.252 a	1.020c	0.152b
	N120	1.507ab	4.544bc	2.294ab	3.176ab	1.136ab	0.160ab
	N180	1.506ab	4.633b	2.295ab	3.183ab	1.123b	0.160ab
	N240	1.533a	4.418bc	2.244b	3.129b	1.146ab	0.165a
	N360	1.522a	4.145c	2.263b	3.145b	1.183a	0.161ab
	Mean	1.507	4.664	2.292	3.177	1.122	0.159
	CV	1.461	4.442	1.758	1.160	2.661	3.243
Taixue 12 (TX12)	N0	1.402b	4.750a	2.508a	3.361a	1.179a	0.133b
	N120	1.494a	4.032b	2.227b	3.067b	1.225a	0.155a
	N180	1.489a	4.037b	2.214b	3.041b	1.222a	0.154a
	N240	1.491a	4.028b	2.237b	3.068b	1.205a	0.155a
	N360	1.476a	3.967b	2.280b	3.120b	1.239a	0.151a
	Mean	1.471	4.163	2.293	3.131	1.214	0.150
	CV	0.750	4.336	1.721	1.381	3.356	2.399

Different lowercase letters indicate statistically significant differences between different treatments at P < 0.05 level.

**Figure 4:**
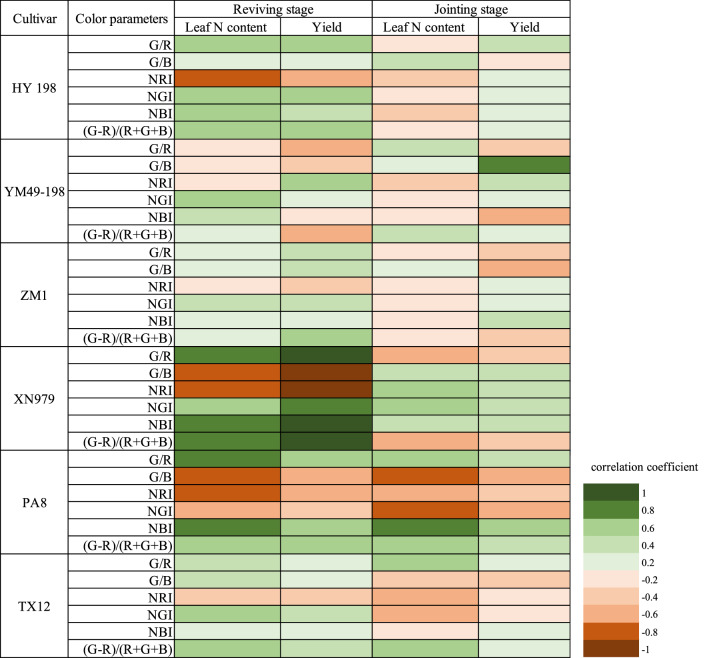
The correlation coefficient between the mean values of the color parameters and leaf N content and yield of different wheat cultivars at different growth stages.

The correlation coefficients between the mean value of the color parameters and leaf N content and yield of the six cultivars were unstable (Fig. 4). At the reviving stage, the correlation coefficients of Huayu 198 (HY198), Xinong 979 (XN979), and PA8 with leaf N content and yield were relatively high and stable. The absolute values of the correlation coefficients between XN979 and yield and leaf N content remained at 0.459–0.694 and 0.745–0.889, respectively. At the jointing stage, the mean value color parameters G/B and NBI of PA8 had the highest correlation coefficients with leaf N content and yield. The correlation coefficients of G/B with yield and leaf N content were − 0.555 and − 0.774, respectively. In addition, from the reviving stage to the jointing stage, the correlation between the mean value of the color parameters and the leaf N content and yield of the six cultivars showed a downward trend.

### Histogram of canopy image color parameters and its relationship with leaf N content and yield

There was a close relationship between the histogram of the color parameters and leaf N content. In the histogram of [(G−R)/(R + G + B)], as an example (Fig. 2a,b), there are two peaks in the histogram that correspond to the dark region and light region in the [(G−R)/(R + G + B)] image. Peak *a* is on the left in the histogram, indicating that this peak represents the proportion of soil pixels in the image. On the contrary, peak *b*, the right peak, represents the light part of the image, which is the crop in the image (Fig. 2a,b). The histogram curves indicated that with the increase in leaf N content, peak *b* in the histogram increased and peak *a* decreased. This trend indicates that the better the crop growth, the larger the proportion of vegetation in the image, with a relative decrease in bare soil. The histogram can not only express the overall color information of the vegetation (peak *b* displacement) but also show the growth information of the vegetation leaves (the relative height of peak *b* and peak *a*). Furthermore, the histograms of the different wheat cultivars showed great differences in both low (Fig. 2c) and high (Fig. 2d) leaf N content.

**Figure 5:**
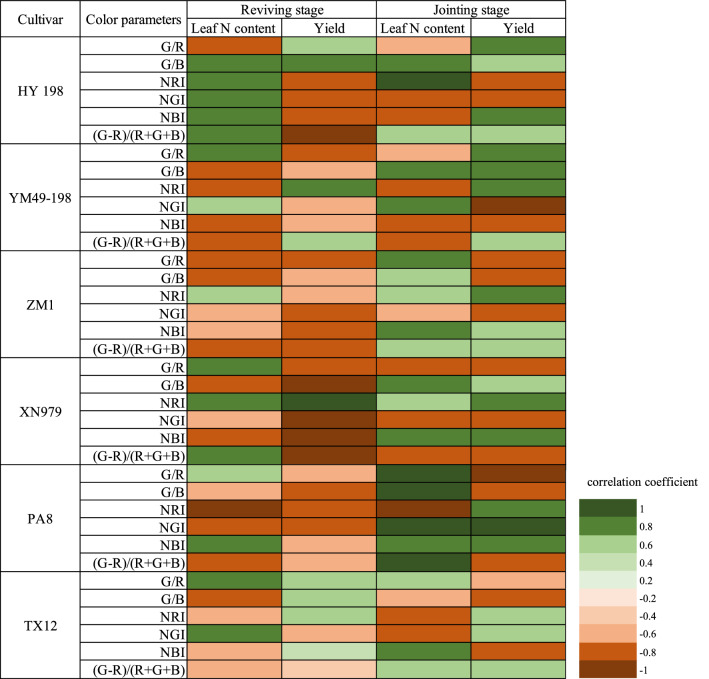
Correlation coefficients of the color parameters histogram and leaf N content and yield of different wheat cultivars at different growth stages.

At the reviving stage and jointing stage of winter wheat, the histograms of the color parameters and leaf N content and yield were analyzed by univariate linear regression. It can be seen from Fig. 5 that the correlation between the color parameter histogram and the leaf N content and yield of the different cultivars was more stable. At the reviving stage of winter wheat, NRI of PA8 had a high negative correlation with leaf N content, with a correlation coefficient of -0.844. At the jointing stage, the [(G-R)/(R + B + G)] of PA8 was positively correlated with leaf N content, with a correlation coefficient of 0.920. The correlation coefficient between the color parameter histogram of PA8 and leaf N content was greater than that of the other cultivars, and the absolute value of the correlation coefficient with yield was 0.679–0.811.

### Comparison of exponential image mean value and exponential image histogram on leaf N content and yield expression ability

**Figure 6:**
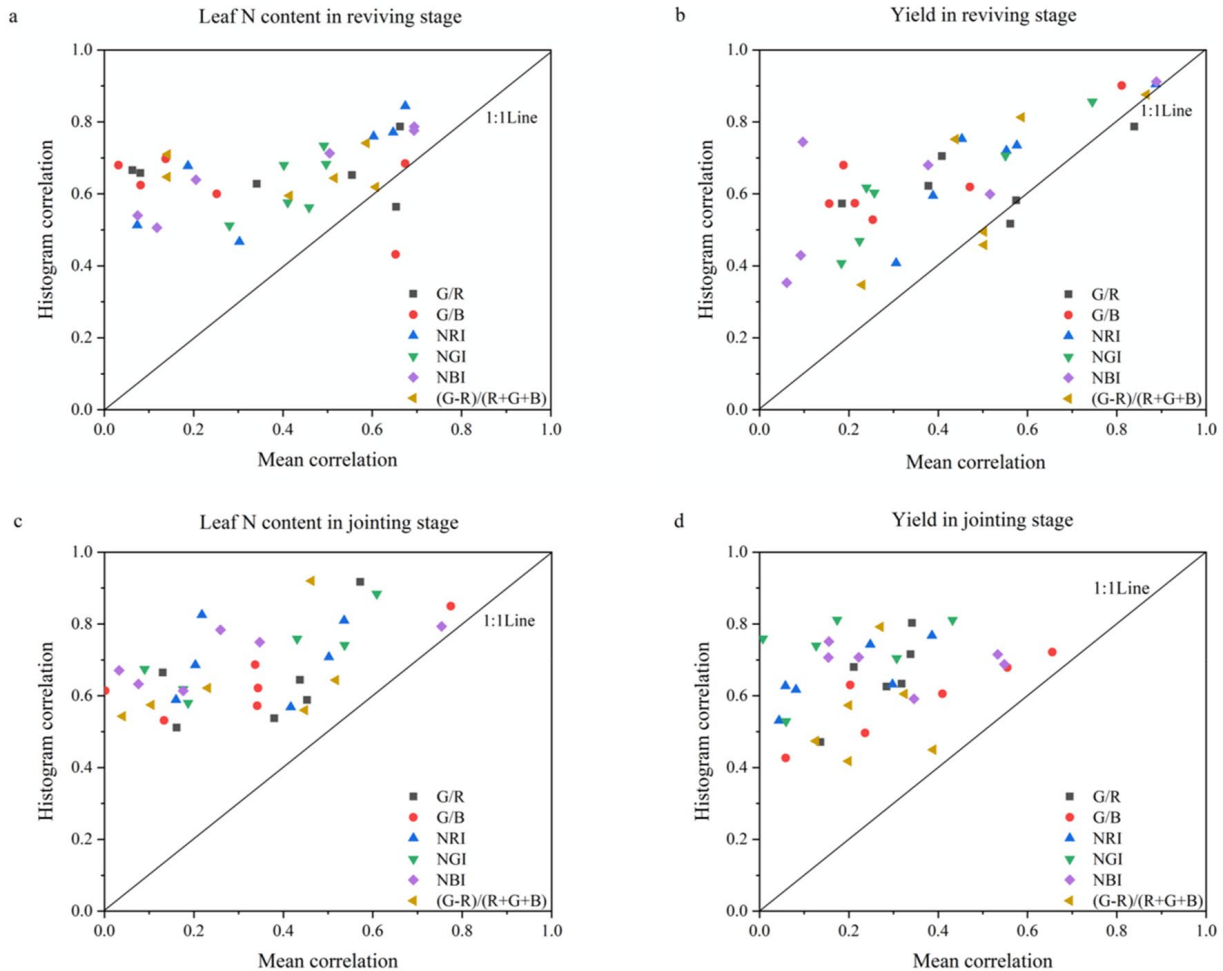
Comparison of image mean value correlations and histogram correlations.

The correlation coefficients of IIMV and IIH in leaf N content and yield were plotted as scatterplots to compare the expression of IIMV and IIH (Fig. 6). It can be seen from Fig. 6a,b that at the reviving stage, the correlation coefficient between IIH and leaf N content and yield was mostly higher than that of IIMV. The scatter points in the graph were all concentrated above the 1:1 line at the jointing stage (Fig. 6c,d). When the correlation coefficient of IIMV was low, the correlation coefficient between IIH and leaf N content and yield was about 0.6. Therefore, IIMV has a better ability to express leaf N content and yield, indicating that compared with IIMV, the histogram features were more accurate in describing the vegetation growth conditions.

### Application of a neural network model to compare exponential image mean value and exponential image histogram

**Figure 7:**
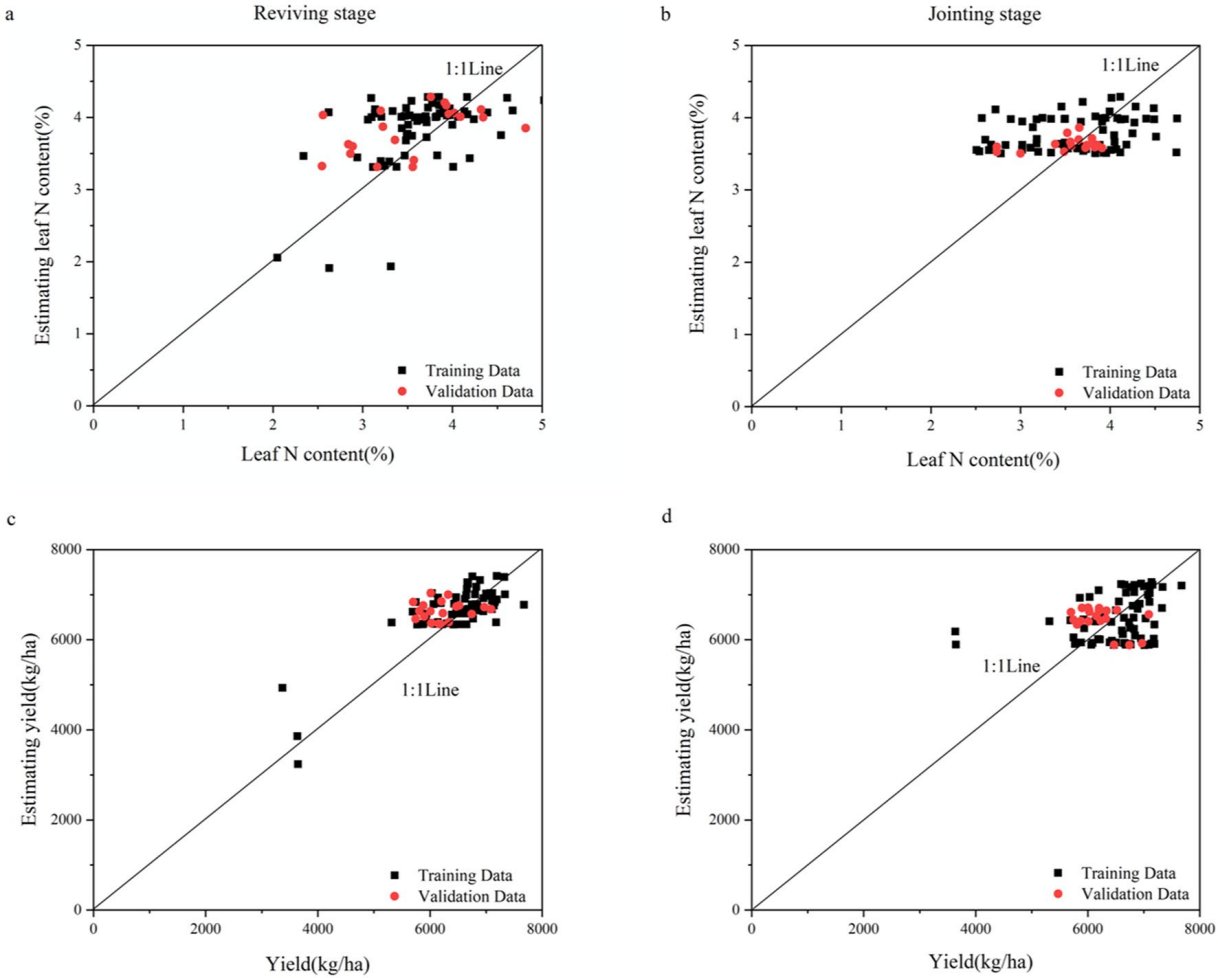
The relationship between the estimated leaf N content and yield and measured values based on the mean value image of wheat.

**Figure 8:**
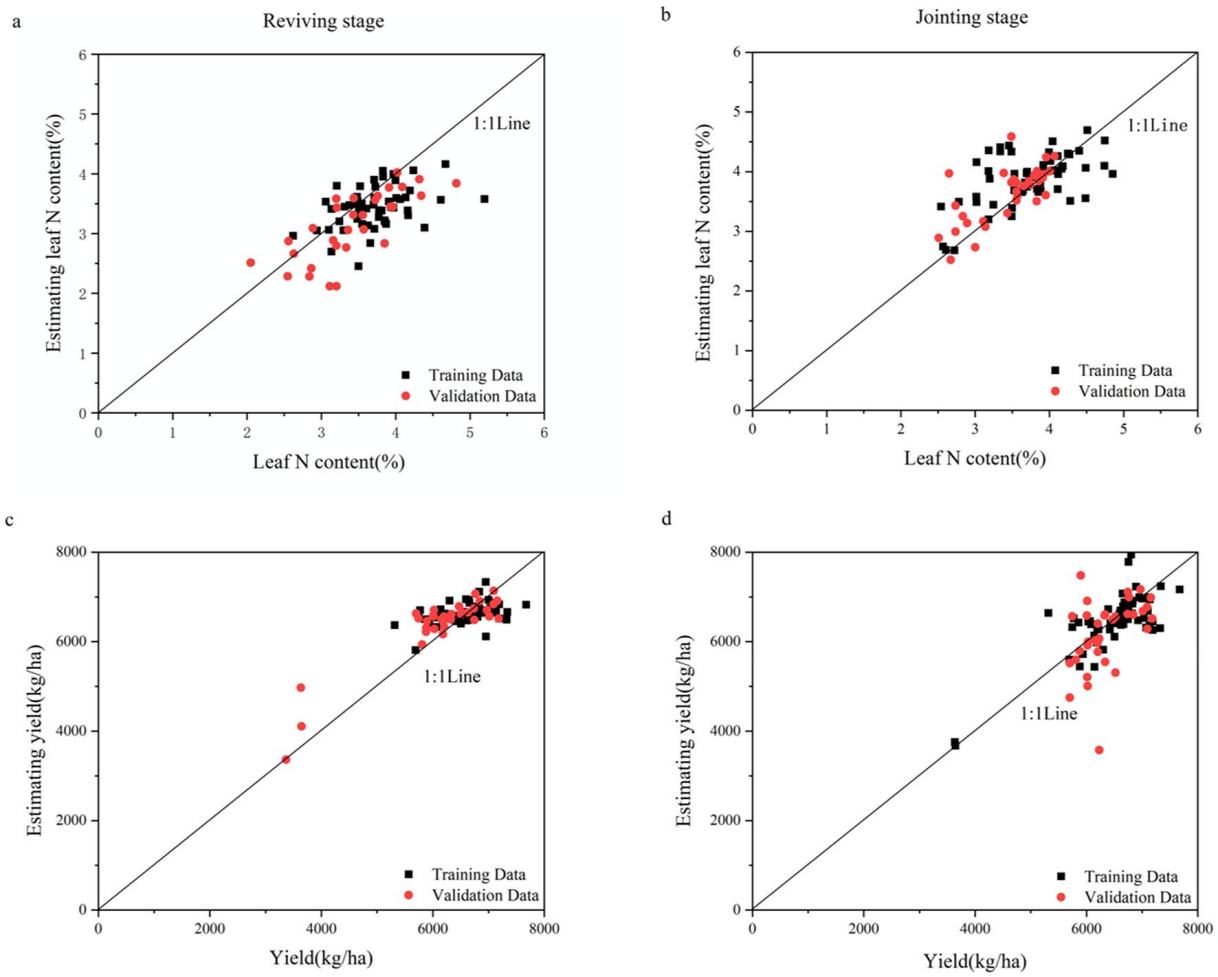
The relationship between the estimated leaf N content and yield and the measured values based on the histogram of the wheat canopy image.

The leaf N content and yield of IIMV and IIH were predicted using a multilayer perceptron (MLP) neural network model. Comparing the estimated and measured values of IIMV and IIH, it can be seen that most of the values were concentrated near the 1:1 line (Figs. 7, 8). After analyzing the error of the neural network, it was concluded that the RMSE and MAPE of IIMV were smaller in the training dataset during the reviving stage (Table 4). This shows that the IIMV training data had a smaller dispersion during the reviving stage of wheat, and the results are thus better. Combining the training dataset and the validation dataset, it is evident that the yield estimation results of IIH were better in the reviving stage (Fig. 8d). However, the leaf N content of IIH had a smaller prediction error and dispersion at the jointing stage. The MAPE and RMSE of IIH were lower than those of IIMV, and the results showed that IIH had a better application effect for different wheat cultivars.

**Table 4:** Comparison of neural network model errors.

Model	Growth stage	Indicators	Training data		Validation data	
			MAPE	RMSE	MAPE	RMSE
Canopy color parameter mean value	Reviving stage	Leaf N content	12.57%	0.551	13.69%	0.553
		Yield	6.34%	487.808	6.88%	526.051
	Jointing stage	Leaf N content	14.04%	0.583	9.91%	0.416
		Yield	8.91%	702.486	9.17%	657.724
Canopy color parameter histogram	Reviving stage	Leaf N content	10.74%	0.522	10.97%	0.499
		Yield	5.94%	460.539	5.00%	393.611
	Jointing stage	Leaf N content	10.60%	0.513	6.27%	0.336
		Yield	5.78%	522.695	5.27%	483.630

## Discussion

### Differences in the images of different wheat cultivars

Differences in the N supply of crops largely affect their leaf N content, which, in turn, results in variations in chlorophyll content and thus leaf color and ultimately plant growth. In contrast to ground hyperspectral devices, multispectral unmanned aerial vehicle sensors, and other high accuracy optical sensors, the diagnosis of N nutrition by a mobile phone camera has the advantages of being convenient and low cost, and has thus gradually attracted the attention of scholars both locally and globally^36–39^. However, the mobile phone camera is not designed for scientific research. Its radiometric sensitivity, spectral response, and signal-to-noise ratio are insufficient in comparison with the precision optical instruments mentioned above. Without the narrow spectral bands and radiometric accuracy, the color indexes from a mobile phone camera developed using remote sensing techniques can only reflect the basic RGB color characters of the winter wheat canopy. Other factors such as leaf coverage, which is also an important indicator for N nutrition, will be neglected when IIMV is used. Thus, the different plant growth statuses at different growth stages will directly affect the performance of IIMV models. If a specific model needs to be built for each wheat cultivar, the mobile phone diagnostic method will be difficult to use at a large scale. Previous studies have indicated that the correlation between canopy color information and chlorophyll content differs greatly for different wheat cultivars (Aozao 8, Hengguan 35, Xinmai 19, Puzhan 4110, Yumai 49-198, and Zhengmai 366)^40^. It can be seen from the image (Fig. 3) that there are some differences among different cultivars at the same stage and under the same treatment. This research supports those results based on comparisons of six wheat cultivars. The correlation between the mean value of the image color parameter at the reviving stage and leaf N content and yield of HY198, XN979, and PA8 was greater than the other cultivars, while at the jointing stage the correlation of PA8 was higher than the other cultivars. The differences between different wheat cultivars may result from three aspects. First, different wheat cultivars have very different leaf color characteristics, and different cultivars may have different canopy structures^11^. Furthermore, changes in the color of wheat leaves can occur when crops suffer from pests and diseases, or when drought occurs. Second, the plant type and height of the different wheat cultivars may cause different reflection curves of the canopy spectrum in the visible light region, which is related to the acquisition of the color parameters of the canopy images^41^. In addition, the growth stage of wheat also affects its dry mass and nutrient accumulation and phenotypic characteristics, leading to canopy image differences, thus affecting the stability and accuracy of the model^42^. For example, in the jointing stage of wheat, wheat growth and nutrient transport are more active, and N accumulation in the plant is in flux, which may affect the accuracy of leaf N concentration measurements and the stability of the canopy image color parameters. In conclusion, the different responses of canopy image color to the wheat cultivars are a disadvantage of the IIMV models.

### Possibility of applying IIH to different mobile phones

Different mobile phones vary in spectrum reflecting, exposure, color tuning, et al. The robustness of the proposed algorithm over different mobile phones is important. In order to verify the application effect of IIH under different mobile phone cameras, we set an independent experiment. In one quadrat, winter wheat canopy images were captured by Apple phone and Meizu phone, respectively. Different from the experiment described in previous sections, there were totally 4 samples collected in this section. The histogram of different mobile phone captured images were calculated and be input into the built network. The MAPE and RMSE indexes from Apple phone and Meizu phone were compared in Table 5. As can be seen from Table 5, in the prediction results of Leaf N content, both the MAPE and RMSE of Apple mobile phone are smaller than those of Meizu mobile phone. Their performance difference is not obvious. An interesting phenomenon is, when estimating yields, Meizu' result is even slightly better than Apple's. Therefore, from the current experiment, the proposed IIH algorithm has great potential in applying to different brands of mobile phones.

**Table 5:** Error comparison of neural network models of different mobile phone images.

Mobile phone brand	Indicators	MAPE	RMSE
Apple mobile phone	Leaf N content	29.68%	1.264
	Yield	7.17%	771.494
Meizu mobile phone	Leaf N content	41.67%	1.68
	Yield	6.31%	604.35

In the future, it is still necessary to make a thorough comparison between different mobile phone brands and even different brand series of cameras to make this technique available in everyday life.

### Advantage of the IIH model

At present, it is necessary to address the stability of the diagnosis model for N nutrition diagnosis using a mobile camera. By taking a canopy image using a mobile phone camera, Xia et al.^42^ found that the visible-light atmospheric-impedance vegetation index (VARI) was significantly correlated with the traditional diagnostic indicator SPAD value and stem base nitrate at the jointing stage of wheat. However, Guo et al.^4^ found that [G/(R + G + B)] had a strong correlation with the leaf N content of maize, thus establishing a maize leaf N detection model. In this study, the results indicated that the single-variable leaf N content and yield estimation models based on IIMV were not stable. The possible reason for this instability is that the mean value of the image color parameters can only reflect the leaf color differences. The plant growth status, which is also an important nutritional status indicator, could not be separated by the single variable. Meanwhile, the method based on the IIH could capture both the color information and the leaf coverage status. The correlation coefficients between the color parameter histograms, leaf N content, and yield of the various cultivars were relatively stable and were significantly higher than the mean value. With more information, a multi-variable model could be built based on the IIH. The experiment results indicated that the IIH multi-variable model could yield stable estimation results depending on the strong nonlinear mapping ability of the neural network algorithm.

From the histogram of the canopy image color parameters based on [(G-R)/(R + G + B)], it can be seen that the peak *b* height of the histogram increased with increasing leaf N content at the reviving stage of wheat, and the two peaks of the different cultivars had different heights. Based on the correlation between canopy color parameter histogram and leaf N content and yield, it is also evident that compared with IIMV, the difference between different cultivars is relatively small. The neural network model was used to test the IIMV model and the IIH model. The vegetation color information and vegetation growth information contained in the IIH model could reduce the differences between the different cultivars. Therefore, in IIH multi-variety N nutrition diagnosis, it is recommended that the IHV model is selected for canopy color parameter histogram construction. In order to carry out nutrition diagnosis more conveniently, it will have a broader prospect to use digital photos for crop N nutrition diagnosis. This paper focuses on explaining the advantages of histogram in nutrition diagnosis, but there are still many problems to be solved. Different brands of mobile phones have various cameras, and there are differences in color temperature, tone and response band of cameras. Therefore, further research is needed to explore the stability and robustness of this method applied to different mobile phone brands. In addition, some alternative techniques such as deep learning neural networks^43^ and energy curve image threshold technique^44^ also have great prospect in digital image processing. Combined with these techniques, the proposed diagnosis structure may be more effective and applicable in the future.

## Conclusions

RGB images of the canopies of six wheat cultivars grown at different N supplies were taken with a smartphone camera during the reviving stage and the jointing stage. From the obtained results, the following conclusions were drawn.

1. The histogram of the color parameters of the wheat canopy images contained abundant information on the growth status of wheat and sufficiently displayed the overall color information and leaf growth. The IIH multi-variable model had a higher correlation with leaf N content and wheat yield than IIMV.
2. In the neural network model, the histogram of the color parameters of the canopy image also produced satisfactory results, and the estimation accuracy and error were better than the parameter mean value method.
3. The histogram of the color parameters of the canopy image combined with the neural network model has strong application potential in the use of mobile phones for the N nutrition diagnosis of wheat.
4. Further study the image analysis technology and deep learning neural network technology, and explore the effectiveness of image threshold setting technology based on energy curve.

## Data Availability

The datasets used and analysed during the current study are available from the corresponding author on reasonable request. All data generated or analysed during this study are included in this published article [and its supplementary information files]. Source data are provided with this paper.
